# Knowledge and Awareness of Stroke among the Elderly Population: Analysis of Data from a Sample of Older Adults in a Developing Country

**DOI:** 10.3390/medicina59122172

**Published:** 2023-12-14

**Authors:** Fouad Sakr, Jihan Safwan, Michelle Cherfane, Pascale Salameh, Hala Sacre, Chadia Haddad, Sarah El Khatib, Mohamad Rahal, Mohammad Dia, Ahmad Harb, Hassan Hosseini, Katia Iskandar

**Affiliations:** 1 School of Pharmacy, Lebanese International University, Beirut 1105, Lebanon; jihan.safwan@liu.edu.lb (J.S.); mohamad.rahal@liu.edu.lb (M.R.); 11410102@students.liu.edu.lb (M.D.); 11231082@students.liu.edu.lb (A.H.); katia_iskandar@hotmail.com (K.I.); 2 UMR U955 INSERM, Institut Mondor de Recherche Biomédicale, Université Paris-Est Créteil, 94010 Créteil, France; hassan.hosseini@aphp.fr; 3 École Doctorale Sciences de la Vie et de la Santé, Université Paris-Est Créteil, 94010 Créteil, France; 4 Institut National de Santé Publique, d’Épidémiologie Clinique et de Toxicologie-Liban (INSPECT-LB), Beirut 1103, Lebanon; michellecherfane@gmail.com (M.C.); pascalesalameh1@hotmail.com (P.S.); halasacre@hotmail.com (H.S.); chadia_9@hotmail.com (C.H.); khatibsarah85@gmail.com (S.E.K.); 5 Gilbert and Rose-Marie Chagoury School of Medicine, Lebanese American University, Byblos 4504, Lebanon; 6 College of Health Sciences, Abu Dhabi University, Abu Dhabi 25586, United Arab Emirates; 7 Faculty of Pharmacy, Lebanese University, Beirut 1103, Lebanon; 8 Department of Primary Care and Population Health, University of Nicosia Medical School, 2417 Nicosia, Cyprus; 9 Research Department, Psychiatric Hospital of the Cross, Jal Eddib 1525, Lebanon; 10 School of Health Sciences, Modern University for Business and Science, Beirut 7501, Lebanon; 11 Faculty of Public Health, Lebanese University, Tripoli 1300, Lebanon; 12 Service de Neurologie, Hôpital Henri Mondor, AP-HP, 94010 Créteil, France

**Keywords:** stroke, knowledge, awareness, senior citizens, elderly, Lebanon

## Abstract

*Background and Objectives:* Stroke prevention has traditionally concentrated on research to improve knowledge and awareness of the disease in the general population. Since stroke incidents increase with age, there is a need to focus on the elderly, a high-risk group for developing the disease. This study aimed to examine the level of stroke awareness and knowledge, their predictors, and their source of information. *Materials and Methods:* A prospective cross-sectional study targeted Lebanese senior citizens aged 65 years and above. A total of 513 participants enrolled in the study through a self-administered survey distributed using a snowball sampling technique. *Results:* Most participants had appropriate baseline knowledge (more than 75% correct answers) of stroke, including risk factors, alarming signs, and preventive measures. Better knowledge of disease risks was significantly associated with having a university degree (ORa = 1.609; *p* = 0.029). Participants who had previous ischemic attacks showed significantly lower knowledge of the alarming signs (ORa = 0.467; *p* = 0.036) and prevention measures (ORa = 0.427; *p* = 0.029). Those suffering from depression had better knowledge of stroke alarming signs (ORa = 2.060.; *p* = 0.050). Seeking information from pharmacists, physicians, or the internet was not significantly associated with better knowledge of stroke risks, alarming signs, and preventive measures. *Conclusions:* The present study showed that seniors had fair knowledge of stroke, despite gaps in stroke prevention measures. Healthcare providers could play a leading role in improving public health by educating seniors to enhance awareness about prevention measures, detecting alarming signs, and acting fast to save a life.

## 1. Introduction

Stroke is a leading cause of morbidity and mortality among the general population [1]. It remains a global health concern that highlights the need for comprehensive stroke risk lowering measures [2]. Education and awareness about the disease are critical for stroke care and prevention approaches [3,4]. Knowledge of stroke has been described as recognizing the risk factors and alarming signs of stroke and responding to its onset appropriately [5,6].

Stroke prevention has traditionally concentrated on research to improve stroke knowledge and awareness among the general population, predominantly those at high risk [7,8]. There is a typical agreement in the literature that stroke knowledge can be enhanced [3], yet there is less agreement on the appropriate way to gauge stroke knowledge levels, and disagreement over who should benefit from this instruction [9]. The results of research looking at how people perceive stroke have been conflicting. While some studies found that people had good knowledge of stroke [7,10], others found a poor understanding of stroke and conveyed a general need for further education [11,12]. The leading cause of these discrepancies could be the difference in geographical regions and variations in the source of information in the studied samples [13]. Mass media, such as television, newspapers, and magazines, are widespread sources of stroke knowledge among the American population [6], with physicians, medical literature, and other sources playing a lower role. Further research among the Australian [5], French [14], and Indian [15] populations also found that professional sources of stroke knowledge, such as physicians, pharmacists, and hospital information, are inferior to electronic and print media. This fact raises serious concerns regarding the quality of public knowledge retrieved from nonprofessional sources and its influence on general knowledge and awareness.

Moreover, published studies on stroke knowledge and awareness encompassed samples from the general population to operationalize stroke knowledge and identify participating individuals who are knowledgeable of stroke. Nevertheless, the risk of stroke correlates with age, with the older population experiencing higher life-threatening incidents [16]. Indeed, around one-third of stroke patients are reportedly elderly, and the risk of death is higher than in younger patients [17]. Studies indicated that senior adults could have reduced stroke knowledge [18,19]. Therefore, a comprehensive evaluation of this population remains warranted to create educational strategies to minimize the stroke burden.

The impact of stroke is higher in developing countries [20], such as Lebanon, a Middle Eastern, lower-middle income Arab country, where stroke is reported to be the second leading cause of death [21]. In Lebanon, which has the highest population aging rate of any Arab nation [22], the prevalence of stroke survivors was estimated at 0.5%, increasing with age to reach 9.38% among those over 80 [23]. Furthermore, risk factors for stroke, including dyslipidemia, diabetes mellitus, hypertension, obesity, and smoking, are highly prevalent in Lebanon [24,25,26]. As a result, the Lebanese population, mainly the elderly, have multiple risk factors for developing stroke.

Strengthening public reaction about the disease is crucial and mandates identifying the gaps in knowledge within the targeted population [27]. Therefore, this study aimed to examine knowledge and awareness of stroke in general, its predictors, and the source of information among a sample of the senior Lebanese population.

## 2. Materials and Methods

### 2.1. Study Design and Participants

This study is part of a larger stroke research project among the senior population. An online cross-sectional study conducted between 1 July 2022 and 30 November 2022 involved 513 Lebanese citizens recruited by snowball sampling. The self-report questionnaire was developed on Google Forms (https://forms.gle/eacoDcJSUheJVTri7) and shared on social media (WhatsApp, Facebook, and LinkedIn). Lebanese senior citizens aged 65 years and above were eligible to participate in the study. Pharmacy students from the Lebanese International University (LIU) participated in data collection and were trained for the questionnaire by one of the investigators of the study to ensure consistency in data collection. Elderly adults with higher levels of education completed the questionnaires with the help of the students, while elderly adults with lower levels of education or illiteracy completed the questions through a structured interview.

### 2.2. Ethical Aspects

The Ethics and Research Committee of the School of Pharmacy at the Lebanese International University approved this study (2020RC-009-LIUSOP). The study was conducted in compliance with the Declaration of Helsinki. Before filling out the online survey, participants were informed about the study objectives and their freedom to withdraw at any time. Participants did not receive any financial reward for their participation. The online survey was anonymous and voluntary. All participants provided informed consent.

### 2.3. Sample Size Calculation

Epi-info software version 7.2.5. was used to calculate the sample size. Considering a population size of 744,590 Lebanese seniors (the elderly constitutes 11% of the population), an expected prevalence of 62.5% of Lebanese participants claiming to know about stroke [28], a 95% confidence level, and a design effect of 1, the minimum sample size was 360.

### 2.4. Online Survey

The online survey tool included closed-ended questions inspired by published articles [29,30,31,32,33,34,35,36,37], and was available in both Arabic and English. The questionnaire consisted of four main sections. The first section covered sociodemographic characteristics (age, gender, marital status, area of residence, education, and health coverage). The second section assessed current health status, including hospitalization in the past six months, history of fall in the past six months, frailty status (assessed using the validated Arabic version of the FRIED score [38]), nutritional status (assessed using the validated mini nutritional score (MNA) [39]), geriatric depression scale (assessed using the validated geriatric depression score (GDS) [40]), the number of comorbidities, polypharmacy (defined by the use of five or more medications, excluding vitamins and minerals), and Body Mass Index (BMI). The third section examined stroke-related knowledge, including where it occurs and its life-threatening nature, lifelong damage, outcomes, risk factors, alarming signs, preventive measures, and appropriate attitude in case of stroke. The fourth part targeted sources of information about stroke, including physicians, pharmacists, and media.

### 2.5. Statistical Analysis

Data were extracted from Google on an Excel spreadsheet and analyzed using SPSS version 25.0. A descriptive analysis evaluated the sample demographic characteristics using the absolute frequencies and percentages for categorical variables and means, and standard deviations (SD) for quantitative measures. Based on the descriptive analysis that showed the percentage of correct and wrong answers about the knowledge of stroke in seniors, data were categorized as less or more than 75% knowledge [41]. For bivariate analysis, the Chi-square test was used to compare the level of knowledge between the three sources of information (physicians, pharmacists, and internet search).

A logistic regression model was conducted, taking the knowledge of stroke risk factors, alarming signs, and preventive measures (less or more than 75%) as the dependent variables. Variables in the model were selected based on the bivariate analysis results with *p*-values < 0.2. These variables included age, gender, university degree, hypertension, transient ischemic attack, hypercholesterolemia, diabetes mellitus, arrhythmia, depression, anxiety, polypharmacy, obesity, number of comorbidities, frailty, physician as a source of knowledge, pharmacist as a source of knowledge, and internet as a source of knowledge.

## 3. Results

### 3.1. Sample Description

Table 1 shows the sociodemographic and other characteristics of the participants. More than half of the participants were females and married, with a mean age of 71.74 ± 6.41 years and a primary education level. Nearly half of the participants had health coverage, 31% reported admission to the hospital in the past six months, 20% had a fall history, 44% took five or more medications, 81% had less than four diseases, 67% had mild depression, 63% were at risk of malnutrition, and 33% were frail. The mean BMI was 22.94 ± 4.27.

### 3.2. Knowledge of Stroke

Table 2 indicates that most participants gave appropriate answers about the nature of stroke and its occurrence, while half did not know that stroke can cause lifelong damage. Less than 75% knew that older age, obesity, transient ischemic attack, diet, and depression increased the risk of stroke. More than 80% knew the alarming signs, preventive measures, and appropriate attitudes in case of stroke, while 58% believed that nothing could be done to prevent stroke.

### 3.3. Sources of Information

Table 3 describes the source of stroke-related knowledge, i.e., from pharmacists, physicians, and the internet. The results showed that a significantly higher proportion of those who knew about the alarming signs of stroke, its lifelong consequences, and the potentially life-threatening nature of this condition received this knowledge from the pharmacist.

Physicians were the source of information for a significantly higher proportion of participants who knew the location of stroke in the body, the alarming signs of stroke, and the appropriate attitude in the event of an incident.

Internet seekers were knowledgeable that stroke is a life-threatening condition and may cause lifelong damage. They also knew that calling the doctor is an appropriate attitude in case of a stroke. However, none of the three sources were significantly associated with better knowledge about the preventive measures for stroke (*p* > 0.05).

### 3.4. Bivariate Analysis

Table 4 indicates that high education levels showed higher knowledge about stroke risk factors and prevention measures. Suffering from diseases such as a previous ischemic attack, depression, or arrhythmia was associated with higher knowledge of stroke alarming signs. Seniors with diabetes mellitus were more knowledgeable of stroke prevention measures. Physicians were a good source of information about stroke risks and alarming signs.

### 3.5. Multivariable Analysis

Table 5 shows the results of three logistic regression analyses, taking knowledge of stroke risk factors, alarming signs, and prevention measures as dependent variables. Variables in the model included gender, university degree, and diseases the patient is suffering from, which constitute an uncontrolled risk factor for stroke, including diabetes mellitus, hypertension, arrhythmia, previous transient ischemic attack, obesity, anxiety, and depression. Other variables were polypharmacy, number of comorbidities, and sources of information (i.e., pharmacists, physicians, or the internet). In each regression, variables were selected based on the bivariate analysis (*p* ≤ 0.2).

In the first logistic regression considering the knowledge about the risk factors of stroke as the dependent variable, the results showed that having a university degree (ORa = 1.609; *p* = 0.029) was significantly associated with a higher level of stroke risk factor knowledge.

The second logistic regression, taking the knowledge about the alarming signs of stroke as the dependent variable, showed that seniors with a history of transient ischemic attack (ORa = 0.467; *p* = 0.036) had a lower level of stroke alarming signs knowledge, as opposed to those who had depression (ORa = 2.060; *p* = 0.050).

In the third logistic regression taking the knowledge about the preventive measures of stroke as the dependent variable, the results showed that seniors with a history of transient ischemic attack (ORa = 0.427; *p* = 0.029) had a significantly lower level of knowledge. Pharmacists, physicians, or the internet as sources of information were not significantly associated with knowledge of stroke risk factors, alarming signs, and prevention measures.

## 4. Discussion

The current study evaluated knowledge and awareness of stroke among a sample of senior adults in Lebanon and found adequate baseline knowledge of stroke. University-educated seniors were more knowledgeable of the risks of stroke, while participants having depression had better knowledge of stroke-associated alarming signs. Poorer knowledge of stroke alarming signs and prevention measures were found in those with a history of transient ischemic attack. This study showed that physicians and pharmacists did not provide adequate knowledge of stroke risks, alarming signs, and prevention measures.

In this study, seniors identified stroke as a life-threatening condition affecting the brain and recognized the alarming stroke signs of sudden onset of confusion, weakness, speech and vision difficulty, severe headache, and loss of balance. Our results indicate a higher level of stroke knowledge among Lebanese senior citizens compared to other regional and international populations [42,43,44]. In 2020, a study reported inadequate knowledge of stroke signs and symptoms among older Lebanese adults in Beirut [28]. The current findings provide better insight into the actual level of knowledge of stroke among senior citizens nationwide as our sample included participants from all over the Lebanese districts, not only from the capital city Beirut.

Previous reports have documented a variable level of knowledge of stroke risk factors [45,46,47,48]. The reason for this discrepancy was the differences in the types of questions about risk factors [49]. The present study included specific closed-ended questions to minimize any possible risk of information bias that might lead to an over- or underestimation of the actual level of knowledge. Participants had fair knowledge of stroke risk factors and could identify most stroke risk factors, including age, lifestyle, and comorbidities. Nevertheless, the gap was at the level of knowledge related to stroke preventive measures, predominantly the role of diet and nutritional behaviors as a risk for stroke. A considerable proportion of participants (45%) had the wrong information about the relationship between eating sweets, fried food, and fatty meals and an increased risk of stroke. Therefore, the current findings warrant additional educational programs to raise awareness that the incidence of stroke decreases with better nutrition and adherence to the appropriate diet recommendations [50].

Participants with a university degree were significantly more knowledgeable of stroke risk factors, consistent with other local and global findings showing that a higher education level is associated with better knowledge of stroke [51,52,53]. Furthermore, a higher level of education was positively correlated with stroke prevention. People with higher education have better adherence to medications used to treat or prevent diseases associated with a higher risk of stroke, including uncontrolled diabetes mellitus, hypertension, dyslipidemia, and atrial fibrillation [54,55,56].

In this study, seniors with depression had better knowledge of stroke alarming signs. This association is not fully understood and was only examined in the post-stroke phase [57]. It is hypothesized that depressed elderly patients are more concerned about their health and tend to seek more information about possible health issues. Nevertheless, depression could also be due to excess illness and anxiety in elderly patients who are more worried about health complications and life [58]. Further studies are needed to examine the relationship between depression and stroke awareness in stroke-naïve patients.

A history of a transient ischemic attack was significantly associated with poorer knowledge of stroke alarming signs and preventive measures. To the best of our knowledge, no prior studies have assessed this association. However, it was previously determined that patients have better stroke knowledge when they have one or more stroke risk factor(s) [59]. Transient ischemic attack is a critical risk factor for recurrent ischemia and stroke [60]. Thus, patients with a history of transient ischemic attack are anticipated to have better knowledge of the alarming signs and possible measures to prevent future strokes. Our findings do not support this hypothesis, likely due to the confusion between transient ischemic attack and stroke. Patients who previously experienced a transient ischemic attack may underestimate the alarming signs of a stroke episode, as the clinical presentation of transient ischemia tends to be less severe and prominent [61]. The current findings are worrisome and necessitate additional counseling for these patients because transient ischemic attack is an established risk factor for stroke and could be linked to poor long-term outcomes [62].

The present study also assessed the sources of information about stroke and their association with stroke knowledge and awareness. Data are scarce in the literature about the impact of sources of information on stroke knowledge and awareness. People reportedly tend to retrieve health information from the internet, which provides an easy, free, and accessible source of information to the public [63]. The problem with health information retrieved from the internet is that it may be misleading or not accurate, and could negatively affect community health [64]. Therefore, it was expected a priori that better stroke knowledge would be associated with receiving information from healthcare professionals. Surprisingly, the current findings showed that patients who received information from physicians and pharmacists do not have better knowledge of stroke risk factors, alarming signs, or preventive measures. Consequently, the role of physicians and pharmacists in raising awareness about stroke appears limited and unsatisfactory in Lebanon, highlighting the need for action plans in this context to strengthen the role of Lebanese healthcare professionals in promoting stroke knowledge, awareness, and preventive care, particularly among the vulnerable senior population.

### Strengths and Limitations

This study has several strengths. It included a sufficient sample size that allowed for all statistical analyses with adequate power. The sample was recruited from all over Lebanon, which provides some generalizability to the current findings. Data were collected by a survey with specific multiple-choice and closed-ended questions, which minimizes the risk of possible information bias. Nevertheless, a few limitations should be acknowledged. First, the cross-sectional design does not establish temporality, and thus causality cannot be confirmed. Second, the snowball sampling technique may have been associated with a possible risk of selection bias, as it may have directed the sample toward a subgroup of the population that is more educated and knowledgeable about stroke. However, it is believed that the risk of this bias is minimized as the sample included participants from all over Lebanon. Finally, residual confounding related to the extent and frequency of stroke counseling by healthcare providers, or by consulting other resources, cannot be precluded. Further studies are suggested to minimize the current biases.

## 5. Conclusions

This study revealed fair knowledge and awareness of stroke among the senior citizens of Lebanon regarding basic information, risk factors, and alarming signs. Nevertheless, healthcare professionals, particularly physicians and pharmacists, appear to have a limited and unsatisfactory role in educating patients and raising awareness about stroke. Considerable gaps have been identified among patients with a history of transient ischemic attack. These findings are alarming and warrant targeted counseling to the senior, stroke-vulnerable population for better awareness, prevention, and outcomes. Additional counseling to the elderly about the relationship between nutrition and stroke and the importance of adhering to dietary recommendations for stroke prevention is also recommended. Moreover, better stroke knowledge and awareness were linked to higher education and depression. Further studies about mental health are recommended in stroke-free patients for a better understanding of this association.

## Figures and Tables

**Table 1:** Sociodemographic characteristics.

Variable	Frequency	%
**Gender**		
Male	245	48
Female	268	52
**Marital status**		
Single/Widowed/Divorced	173	34
Married	340	66
**Working region**		
Beirut/ Mount Lebanon/ Beqaa	214	42
North and South of Lebanon	299	58
**Health coverage**		
Private or public insurance	286	56
No health coverage	227	44
**Educational level**		
Primary education	287	56
Secondary or tertiary education	226	44
**Admission to the hospital in the past six months**		
No	354	69
Yes	159	31
**Fall history in the past six months**		
No	412	80
Yes	101	20
**Polypharmacy**		
No	290	56
Yes	223	44
**Number of comorbidities**		
Less than four diseases	416	81
Four diseases or more	97	19
**Geriatric Depression Scale**		
Normal	34	7
Mild depression	342	67
Moderate depression	120	23
Severe depression	17	3
**Frailty**		
Non frail	89	17
Pre-frail	252	50
Frail	172	33
**Nutritional status**		
Malnourished	91	18
At risk of malnutrition	322	63
Normal nutritional status	100	19
	**Mean**	**SD**
**Age**	71.74	6.41
**BMI (Body Mass Index)**	22.94	4.27

**Table 2:** Knowledge of stroke risk factors, alarming signs, preventive measures, and appropriate attitudes in case of an event.

Variable	Wrong Answer		Right Answer	
	n	%	n	%
Stroke is a life-threatening condition	71	14	442	86
Stroke occurs in the brain	106	21	407	79
Stroke can cause lifelong damage	259	51	254	49
Risk factors of stroke				
**Health status**				
Uncontrolled hypertension	48	9	465	91
Uncontrolled diabetes	183	36	330	64
Arrythmia	133	26	380	74
Obesity	148	29	365	71
Previous transient ischemic attack	184	36	329	64
Previous stroke/Family history of stroke	67	13	446	87
High cholesterol and triglycerides levels	119	22	400	78
Advanced age	149	29	384	71
**Lifestyle**				
Eating sweets and fried food and fatty meals	231	45	282	55
Not eating enough fruits and vegetables	154	30	359	70
Poor diet/Not eating well	204	40	309	60
Depression	217	42	296	58
Lack of exercise	136	26	377	74
Drinking alcohol	132	26	381	74
Smoking	119	23	394	77
**Alarming signs of stroke**				
Sudden onset of weakness or numbness on one side of the body	64	12	449	88
Sudden speech difficulty or confusion	67	13	446	87
Sudden difficulty seeing in one or both eyes	88	17	425	83
Sudden onset of dizziness, trouble walking, or loss of balance	78	15	435	85
Sudden, severe headache with unknown cause	85	17	428	83
**Preventive measures of stroke**				
Regular exercise	60	12	453	88
Stop drinking alcohol	63	12	450	88
Stop smoking	77	15	436	85
Managing diabetes	106	21	407	79
Eating healthy diet	65	13	448	87
Medication use for the prevention of stroke such as aspirin	73	14	440	86
Managing heart disease	79	15	434	85
Managing hypertension	45	9	468	94
Treating high blood cholesterol and triglycerides	74	14	439	86
Reducing stress	57	11	456	89
There is nothing to be done to prevent stroke	297	58	216	42
**Appropriate attitude in case of an event**				
Take the patient to the hospital	50	10	463	90
Call the doctor	126	25	387	75
Call an ambulance	36	7	477	93

**Table 3:** Sources of information about stroke.

Variable	Pharmacists				Physicians				Internet Search			
	N = 290				N = 382				N = 210			
	n	%	n	%	n	%	n	%	n	%	n	%
	Yes		No		Yes		No		Yes		No	
**Knowledge of the location of stroke occurrence in the body**												
No	176	79	47	21	94	72	37	28	243	80	60	20
Yes	231	80	59	20	313	82	69	18	164	78	46	22
*p* value	0.462				0.01				0.319			
**Knowledge that stroke is a life-threatening condition**												
No	199	89	24	11	115	88	16	12	269	89	34	11
Yes	243	84	47	16	327	86	55	14	173	82	37	18
*p* value	0.049				0.321				0.027			
**Knowledge that stroke can cause lifelong damage**												
No	121	54	102	46	100	76	31	23	119	39	184	61
Yes	133	46	157	54	154	40	228	60	135	64	75	36
*p* value	0.036				<0.001				<0.001			
**Knowledge of the risk factors of stroke**												
No	122	55	101	45	80	61	51	39	168	55	135	45
Yes	158	54	132	46	200	52	182	48	112	53	98	47
*p* value	0.516				0.052				0.351			
**Knowledge of the alarming signs of stroke**												
No	165	74	58	26	92	70	39	30	236	78	67	22
Yes	241	83	49	17	314	82	68	18	170	81	40	19
*p* value	0.008				0.003				0.233			
**Knowledge of the preventive measures of stroke**												
No	157	70	66	30	97	74	34	26	216	71	87	29
Yes	210	72	80	28	270	71	112	29	151	72	59	28
*p* value	0.343				0.268				0.480			
**Knowledge about the appropriate attitude in case of stroke**												
Take the patient to the hospital												
No	197	88	26	12	111	85	20	15	269	89	34	11
Yes	266	92	24	8	352	92	30	8	194	92	16	8
*p* value	0.129				0.013				0.114			
**Call the doctor**												
No	161	72	62	28	94	72	37	28	216	71	87	29
Yes	226	78	64	22	293	77	89	23	174	81	39	19
*p* value	0.082				0.155				0.005			
**Call an ambulance**												
No	203	91	20	9	115	88	16	12	278	92	25	8
Yes	274	94	16	6	362	95	20	5	199	95	11	5
*p* value	0.090				0.008				0.127			

**Table 4:** Bivariate analysis of stroke risk factors, alarming signs, and prevention measures.

Variables		Knowledge of Stroke Risk Factors			Knowledge of Stroke Alarming Signs			Knowledge of Stroke Prevention Measures		
		n	%	*p*-Value	n	%	*p*-Value	n	%	*p*-Value
Gender	Female	147	52	0.484	210	52	0.364	199	54	0.092
	Male	133	48		196	48		168	46	
University degree	Yes	76	27	0.052	104	26	0.085	97	26	0.036
	No	204	73		302	74		270	74	
History of transient ischemic attack	Yes	57	10	0.123	28	7	0.035	27	7	0.181
	No	253	90		378	93		340	93	
Having Diabetes Mellitus	Yes	122	44	0.517	176	43	0.431	149	41	0.017
	No	158	56		230	57		218	59	
Diagnosed with anxiety	Yes	91	33	0.060	147	36	0.355	123	33	0.065
	No	189	67		259	64		244	66	
Diagnosed with depression	Yes	42	15	0.492	68	17	0.036	55	15	0.462
	No	238	85		338	83		312	85	
Polypharmacy	Yes	120	43	0.414	179	44	0.330	156	42	0.274
	No	160	57		227	56		211	58	
Having more than five comorbidities	Yes	53	19	0.541	78	19	0.426	66	16	0.233
	No	227	81		328	81		301	82	
Obesity	Yes	17	6	0.176	21	5	0.533	17	5	0.304
	No	263	93		385	85		350	95	
Having hypertension	Yes	175	62	0.145	247	61	0.331	220	60	0.457
	No	105	38		159	39		147	40	
Having hypercholesterolemia	Yes	140	50	0.177	209	51	0.347	183	50	0.070
	No	140	50		197	49		184	50	
Having arrythmia	Yes	86	31	0.319	137	34	0.038	120	33	0.273
	No	194	69		269	66		247	67	
Physician as a source of information	Yes	200	71	0.052	314	77	0.003	270	74	0.268
	No	80	29		92	23		97	26	
Pharmacist as a source of information	Yes	158	56	0.516	241	59	0.008	210	57	0.343
	No	122	44		165	41		157	43	
Internet as a source of information	Yes	112	40	0.351	170	42	0.233	151	41	0.480
	No	168	60		236	58		216	59	

**Table 5:** Knowledge about stroke risk factors, alarming signs, and prevention measures.

Variables	Beta	*p*-Value	Ora *	95% CI for ORa	
				Lower	Upper
	**Knowledge of stroke risk factors**				
University degree	0.475	0.029	1.609	1.049	2.466
History of transient ischemic attack	0.446	0.200	1.562	0.790	3.090
Diagnosed with anxiety	−0.347	0.071	0.707	0.485	1.030
Obesity	0.661	0.126	1.937	0.830	4.523
Suffering from hypertension	0.385	0.057	1.470	0.989	2.185
Having hypercholesterolemia	−0.250	0.203	0.779	0.530	1.145
Physician as a source of information	−0.391	0.064	0.676	0.447	1.023
	**Knowledge of stroke alarming signs**				
University degree	0.302	0.282	1.353	0.780	2.346
History of transient ischemic attack	−0.761	0.036	0.467	0.230	0.950
Diagnosed with depression	0.723	0.050	2.060	0.999	4.247
Physician as a source of information	0.443	0.112	1.557	0.901	2.690
Pharmacist as a source of information	0.314	0.252	1.369	0.800	2.342
Internet as a source of information	−0.004	0.987	0.996	0.619	1.603
	**Knowledge of stroke prevention measures**				
University degree	0.325	0.244	1.384	0.801	2.393
History of transient ischemic attack	−0.850	0.029	0.427	0.200	0.915
Gender	−0.060	0.788	0.942	0.609	1.458
Having diabetes mellitus	−0.065	0.782	0.937	0.590	1.487
Diagnosed with anxiety	0.073	0.772	1.076	0.655	1.769
Having hypercholesterolemia	−0.141	0.555	0.868	0.543	1.388
Suffering from more than five comorbidities	0.429	0.254	1.536	0.735	3.210

* Knowledge: more than 75% right answers.

## Data Availability

The data presented in this study are available from the corresponding author on reasonable request.
